# Prevention of quality characteristic decline in freeze‐thawed cultured large yellow croaker (*Larimichthys crocea*) using flammulina velutipes polysaccharide

**DOI:** 10.1002/fsn3.3051

**Published:** 2022-10-17

**Authors:** Yuzhuo Shi, Yao Zheng, Baoguo Li, Xu Yang, Quanyou Guo, Anqi Liu

**Affiliations:** ^1^ Ministry of Agriculture and Rural Affairs, East China Sea Fisheries Research Institute Chinese Academy of Fishery Sciences Shanghai China; ^2^ School of Health Science and Engineering University of Shanghai for Science and Technology Shanghai China

**Keywords:** antifreeze property, cultured large yellow croaker, flammulina velutipes polysaccharide, quality characteristics

## Abstract

To investigate the cryoprotective effect of flammulina velutipes polysaccharide (FVP) on the quality characteristics in freeze‐thawed cultured large yellow croaker, 0.050%, 0.075%, and 0.100% FVP was used before freezing and the quality after thawing was compared with water soaking (WS) and commercial cryoprotectant (CC) treatment. Quality attributes were comprehensively determined instrumentally and organoleptically after thawing at 4°C. Results showed that FVP effectively reduces the quality deterioration of body color and water‐holding capacity, while no obvious effects were observed in texture and flavor. As for body color, both FVP and CC treatment could maintain the b* value to a large extent. Among them, 0.075% FVP shows the highest value in two sample points, with 55.2% and 21.0% increases seen in the values in WS. FVP‐dose‐dependent trends were found in water‐holding capacity, where a reduction of 28.26% and 14.38% in thawing loss and cooking loss was observed in the 0.100% FVP group. Low‐field nuclear magnetic resonance (LF‐NMR) also revealed that immobilized water and free water were more tightly retained in the muscle tissue with FVP addition. The results of the sensory evaluation are essentially in line with the above observations. These findings indicate that FVP has the potential to partially replace commercial cryoprotectants in aquatic products during frozen storage.

## Graphical abstract

Our project aims to reveal the cryoprotective effect of flammulina velutipes polysaccharide (FVP) on the quality characteristics in freeze‐thawed cultured large yellow croaker. The differences were compared with conventional commercial cryoprotectant in combination with muscle quality, body color, and flavor. From the point of delaying the deterioration of frozen aquatic products’ quality, we believe that this paper is important for extending the shelf life of frozen aquatic products, and for further development of novel aquatic cryoprotectant.

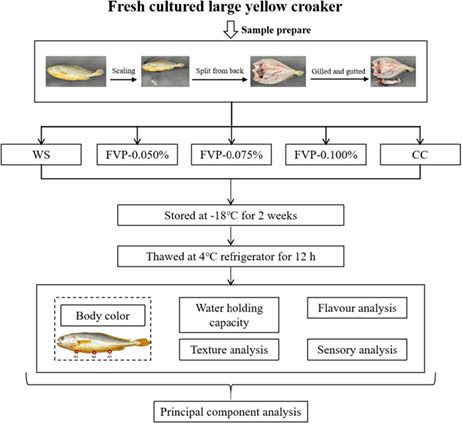

## 1 INTRODUCTION

As one of the most important commercial fish products in China, the large yellow croaker (*Larimichthys crocea*) is highly prized for its nutrition, flavor, and shiny yellow appearance, especially the golden yellow color of its abdomen. The production of this fish reached 25.4 million tons in 2020, a 12.6% increase compared to 2019, meaning that the fish has continuously ranked top of the marine culture fishes in China (Fishery Bureau of Ministry of Agriculture of the People's Republic of China, 2021). Some 90% of the total output of large yellow croaker is transported by ice storage and frozen storage; nowadays, as cold chain logistics continue to improve and the demand for convenience among young consumers continues to rise, the proportion of frozen products rises further still.

Freezing and subsequent frozen storage are the industry standard preservation methods for extending the shelf life of aquatic products because of their effectiveness in inhibiting endogenous enzyme activity and microbial growth (Tolstorebrov et al., 2016; Zheng et al., 2021). However, quality deterioration inevitably occurs in freeze‐thawed aquatic products, leading to both sensory and economic disadvantages (Zhang, Cao, et al., 2019; Zhang, Zhao, et al., 2019). It is generally believed that the growth and recrystallization of ice crystal largely contributed to the quality loss. The formation of ice crystal will mechanically disrupt the cellular structure and cause the release of metals, ions, and enzymes. Meanwhile, the unfrozen water will concentrate the solutes, namely freezing concentration, accelerating the biochemical reaction of protein denaturation and lipid oxidation (Zhang, Cao, et al., 2020). Numerous studies have reported the quality loss of water‐holding capacity, texture, and flavor. Studies have shown that protein denaturation due to protein oxidation during freezing may lead to reduced juiciness and exudation of free water in shrimp (Haghshenas et al., 2015; Shi et al., 2019). In addition, changes in the shape, size, and distribution of ice crystals during storage may lead to changes in the water‐holding capacity and texture of frozen muscle tissue (Zhang, Cao, et al., 2020). Qi et al. (2021) used electronic nose (E‐nose) and electronic tongue (E‐tongue) to evaluate the degree of deterioration in product flavor during frozen storage, while Xu et al. (2020) suggested that assisted sensory evaluation could more directly reflect consumer preferences and may also explain subtle differences that cannot be detected by instruments. It has therefore become imperative to utilize appropriate antifreeze strategies in order to maintain the natural quality of aquatic products such as large yellow croaker.

Cryoprotectants are extensively used in frozen aquatic products in order to minimize quality loss during the freeze–thaw process. A mixture of sucrose and sorbitol had become the customary cryoprotectant due to its great efficiency in reducing ice crystal formation and protein denaturation (Liu et al., 2019). However, its cryoprotective effects were unfortunately accompanied by an excessively sweet taste and higher caloric value, leading to changes in the taste and nutritional value of the product, along with potential health issues (Xiong et al., 2009). Experimental attention therefore turned to finding and developing cryoprotectants with low calorie and low sweetness, namely Konjac glucomannan (Xiong et al., 2009), soluble soybean polysaccharides (Gao et al., 2019), carrageenan oligosaccharides, and other natural polysaccharides (Lan et al., 2020). The ice crystal inhibition effect, cryostabilized effects, and antioxidant activity of polysaccharides have attracted a lot of interest for their antifreezing prospect (Wang & Zhang, 2021).

Flammulina velutipes polysaccharide (FVP) is a water‐soluble polysaccharide extracted from the substrate or mycelium of the flammulina velutipes. It cannot be hydrolyzed by human digestive enzymes. FVP is composed of more than 10 monosaccharides linked by β (1→3) glycosidic bonds. The monosaccharide composition of the FVP as detected by high‐performance gel permeation chromatography is predominantly glucose (70.41%), galactose (16.38%), and mannose (7.74%) (Hao et al., 2021). As one of the main bioactive compounds of the flammulina velutipes, FVP has a variety of functional properties. Its function has been reported in immune regulation, antioxidation, intestinal flora regulation, improvement of memory (Wang & Zhang, 2021), and also the antifreeze property by reducing the formation of ice crystals (Kawahara et al., 2016). However, at this time the effect of its antifreeze property on the quality of aquatic products has received limited attention, especially when applied to large yellow croaker.

To investigate the cryoprotective effect of FVP on the eating quality of cultured large yellow croaker during the freeze–thaw process, the body color, water‐ holding capacity, texture, and flavor are comprehensively analyzed. The aim of this study is to find a novel cryoprotectant for aquatic products and to provide theoretical support for the commercial application of FVP.

## 2 MATERIALS AND METHODS

### 2.1 Sample preparation

**FIGURE 1:**
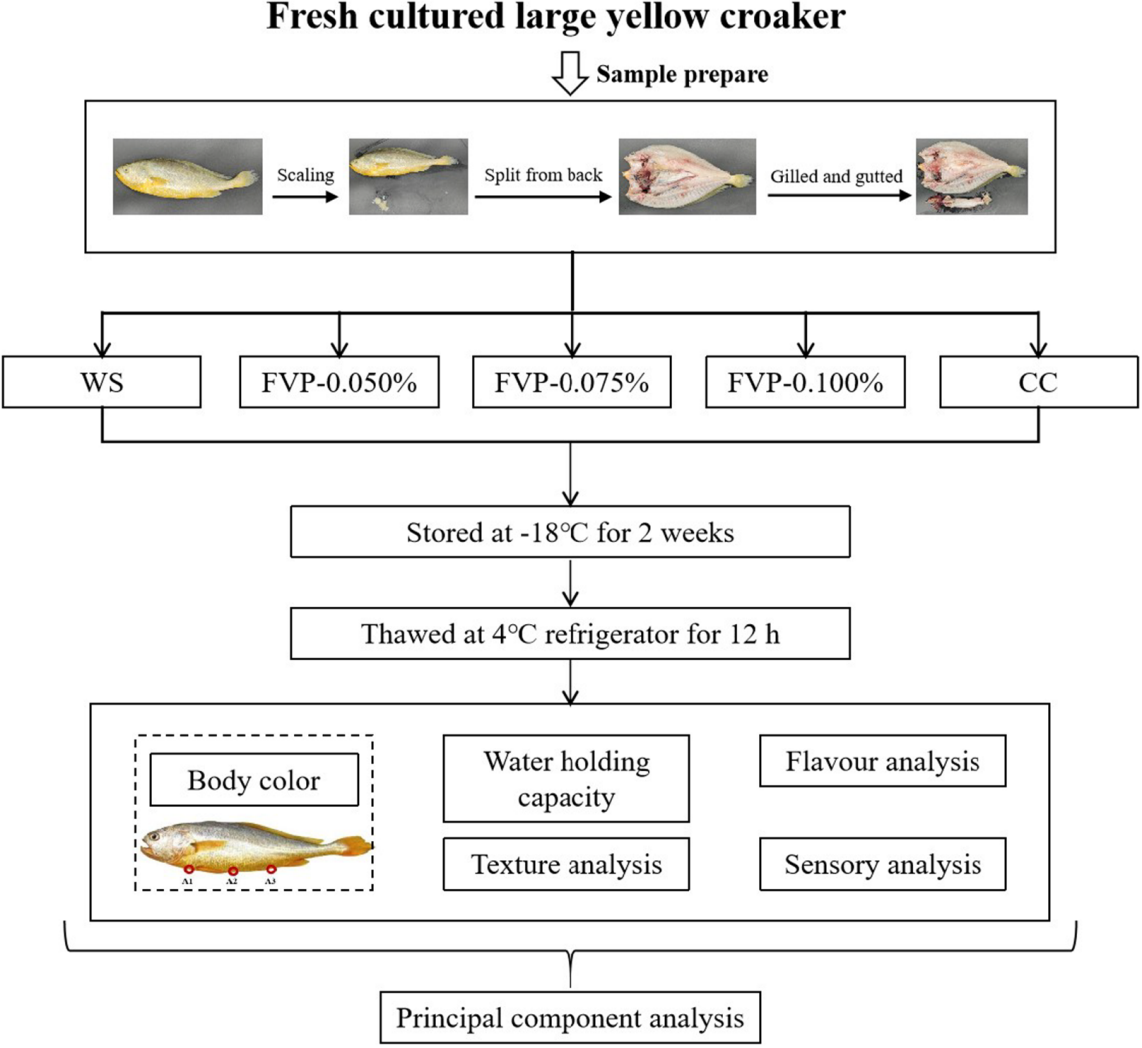
Schematic diagram of experimental steps. CC, samples soaked in commercial cryoprotectant; FVP‐0.050%, samples soaked in 0.050% flammulina velutipes polysaccharide (FVP) solution; FVP‐0.075%, samples soaked in 0.075% FVP solution; FVP‐0.100%, samples soaked in 0.100% FVP solution; WS, samples soaked in distilled water

Fresh large yellow croakers (weight: 488.70 ± 26.62 g; *n* = 30) were purchased whole from a local market in Shanghai and transported within 1 h to the laboratory in an ice‐filled styrofoam box. After arrival, the fish were split along the back, gutted, descaled, and bled. The samples were then randomly divided into five groups: (1) the water soaking group (WS), the blank control group for which the samples were soaked in distilled water; (2) the 0.050% FVP group (FVP‐0.050%) for which the samples were soaked in 0.05% FVP solution (w/w); (3) the 0.075% FVP group (FVP‐0.075%) for which the samples were soaked in 0.075% FVP solution (w/w); (4) the 0.100% FVP group (FVP‐0.100%) for which the samples were soaked in 0.100% FVP solution (w/w); and (5) the commercial cryoprotectant group (CC), the positive control group for which the samples were soaked in 4% sorbitol and 4% sucrose (w/w). All five groups were soaked at 4°C for 1 h, then drained and packed in polyethylene bags before being stored at −18°C for further analysis (Figure 1).

### 2.2 Body color

The skin color of the thawed samples was measured using a Minolta Chroma Meter (CR‐400, Konica Minolta). The three points A1, A2, and A3 from the abdomen of the fish body were selected (Figure 1); L* (brightness), a* (redness), and b* (yellowness) were determined according to the CIE L* a* b* color system, which was calibrated with a white standard plate (L* = 90.26, a* = −1.29, b* = 5.18).

### 2.3 Thawing loss and cooking loss

Water‐holding capacity was evaluated by measuring thawing loss and cooking loss, as described by Tan et al. (2021). The weight of frozen fish before (*M*_1_) and after thawing (*M*_2_) was recorded, and thawing loss was calculated by the following Equation (1):

(1) $$\text{Thawing}\;\text{loss}\left(\%\right)=\frac{\left(M_{1}-M_{2}\right)}{M_{1}}\times 100$$

The weight of thawed fish before (*M*_3_) and after cooking (*M*_4_) was also recorded, with the cooking loss calculated by the following Equation (2):

(2) $$\text{Cooking}\;\text{loss}\left(\%\right)=\frac{\left(M_{3}-M_{4}\right)}{M_{3}}\times 100$$

### 2.4 Low‐field nuclear magnetic resonance (LF‐NMR)

The LF‐NMR was measured as described by Kawahara (2002), with some modifications. The distribution and migration of water were rapidly evaluated with an LF‐NMR analyzer (Suzhou Niumag Analytical Instrument Corporation) with a magnetic field strength of 0.5 T and a proton resonance frequency of 20 MHz. The dorsal muscle samples were cut into 2 cm × 2 cm × 1 cm pieces and covered with plastic wrap. The magnet operating temperature was 32°C, and T_2_ was determined with the Carr–Purcell–Meiboom–Gill (CPMG) pulse sequence. For each measurement, four scans were performed with 5000 echoes.

### 2.5 Shear force and texture profile analysis

Muscle texture measurements were performed according to the method described by Wang et al. (2022), with some modifications. The dorsal muscles were cut from the fish and the skin was removed, then the flesh was cut into 20 mm × 20 mm × 10 mm blocks for analysis. A TMS‐Pro Texture Analyzer (Food Technology Corporation) equipped with a flat‐bottomed cylindrical probe (p/5) and a heavy blade set was used to examine the muscle samples. The trigger force was 5 g and the test speed was 3 mm/s. Compression strain was set to 50%.

### 2.6 Electronic tongue analysis

Flavor characteristics were measured using a TS‐5000Z E‐tongue (Insent), according to the method previously described by Du et al. (2021), with some modifications. Forty grams of minced muscle sample was mixed with 200 ml ultrapure water before being homogenized. The mixed solution was centrifuged at 1089 *g* for 5 min, and the resulting supernatant was then used for analysis through the E‐tongue taste sensing system; the system consisted of six chemical sensors (umami, sourness, saltiness, bitterness, astringency, and sweetness).

### 2.7 Electronic nose analysis

Volatile compounds were measured with the PEN3 (Portable Electronic Nose) (Airsense) using an E‐nose, based on the method previously described by Yang et al. (2020), with some modifications. The PEN3 has an array of 10 different metal oxide sensors, including WC1 (sensitive to aromatic compounds), W5S (sensitive to nitrogen oxides), W3S (sensitive to ammonia, aromatic compounds), W6S (sensitive to hydrides), W5C (sensitive to short‐chain alkanes, aromatic compounds), W1S (sensitive to methyl), W1W (sensitive to sulfides, pyrazine, many terpenes), W2S (sensitive to alcohols, aldehydes, and ketones), W2W (sensitive to organic sulfides, aromatic compounds), and W3S (sensitive to long‐chain alkanes). Ten grams of minced muscle sample was loaded into a 40 ml sample vial and incubated at room temperature for 30 min. The E‐nose system parameters were set with an injection flow rate of 400 ml/min, and the measurement lasted 60 s.

### 2.8 Sensory analysis

Sensory analysis was performed according to the method previously described by Lan et al. (2021), with some modifications. The evaluation was performed by individual judgments in the laboratory, with the sensory analysis of the thawed samples carried out by seven panelists. The weightings for muscular tissue, color, odor, and texture were determined to be 22%, 28%, 25%, and 25%, respectively, based on the importance of large yellow croaker in the sensory evaluation. The panelists marked according to their sensory interpretations in the range of 1–5: color (5 = bright golden; 1 = dull pale), odor (5 = desirable; 1 = extremely unacceptable), texture (5 = firm; 1 = soft), and muscular tissue (5 = clear; 1 = cloudy).

### 2.9 Statistical analysis

All statistical evaluations were expressed as means ± standard deviation (SD). The data were subjected to one‐way analysis followed by Duncan's multiple comparisons test using SPSS 22 to determine significant differences between the groups. The confidence interval (CI) was set at 95%. The correlation of overall differences between groups and quality was used for principal component analysis (PCA) in SIMCA software (version 14.1, Umetrics).

## 3 RESULTS AND DISCUSSION

### 3.1 Body color

**FIGURE 2:**
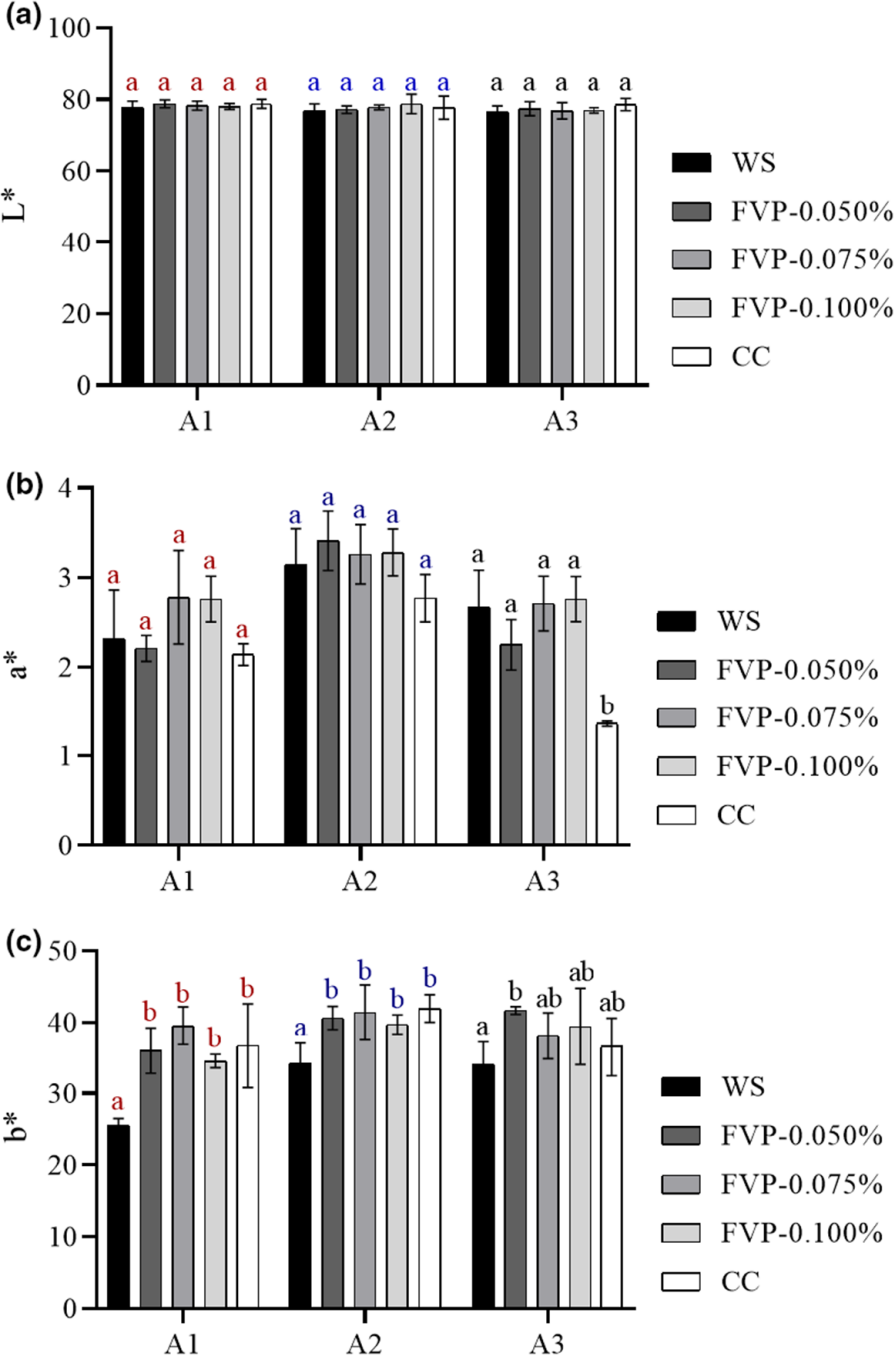
Changes in L* (a), a* (b), and b* (c) values of large yellow croaker with different cryoprotectant treatments. A1, A2, and A3 represent the three different sampling points on the abdomen of the large yellow croaker. CC, samples soaked in commercial cryoprotectant; FVP‐0.050%, samples soaked in 0.050% flammulina velutipes polysaccharide (FVP) solution; FVP‐0.075%, samples soaked in 0.075% FVP solution; FVP‐0.100%, samples soaked in 0.100% FVP solution; WS, samples soaked in distilled water. Different letters (a and b) represent a significant difference between samples at the same sampling point with different treatments (*p* < .05).

Body color is among the most crucial of eating qualities affecting commodity value and consumer acceptance, especially for large yellow croaker (Ma et al., 2021). The change in body color from the abdomen is displayed in Figure 2. Three representative test points out of the 16 points of the whole fish body were selected according to our previous study (Figure 1). In terms of b* value, which stands for the specific yellow color of large yellow croaker, the WS group was significantly lower than those of the other four treated groups among A_1_, A_2_, and A_3_. Among them, the FVP‐0.075% group shows the highest value in both A_1_ and A_2_, with a 55.2% and 21.0% increase in the WS values, respectively. The b* value is highly correlated with the carotenoid content in pigment cells of their skin (Yi et al., 2015), which we presume was disrupted by the mechanical damage induced by the growth of ice crystals (Tan et al., 2021). By inhibiting the growth of these ice crystals, the FVP groups overall and the CC group prevent the descent of the b* value. Additionally, carotenoids are susceptible to oxidative damage due to the composition of highly unsaturated double bonds (Lyu et al., 2021). Zhang et al. 2018 found that FVP has a strong hydrogen‐donating ability and can bind to free radicals to further form stable free radicals and inhibit free radical chain reactions. Therefore, the antioxidant properties shown by FVP can slow down the decrease in b* value to a certain extent (Ruthes et al., 2016), which was supported by the slightly higher b* value of FVP groups than the CC group (which showed no significant difference). As for L* and a* values, almost no significant difference was observed across the different groups. It can thus be seen that FVP can reduce the deterioration of the body color in freeze‐thawed cultured large yellow croaker, with effectiveness similar to the existing commercial cryoprotectant agent.

### 3.2 Texture analysis

Texture attributes are another critical eating quality, and are thus deserving of more attention in aquatic product storage due to the undesired “softening” or “weakening” phenomenon (Jiang et al., 2022). The results of our texture profile analysis and shear force are shown in Table 1. Compared with the WS group, the CC group showed a significant increase in the value of hardness, springiness, gumminess, and chewiness (*p* < .05), which was similar to results seen in trout fillets (Jittinandana et al., 2004). An increasing trend was observed in all three FVP groups across the four textural attributes described above, but without significant difference. As for shear force, used as the indicator for muscle tenderness, higher values were found in the CC group and FVP groups, with the largest being the 20.1% increase seen in FVP‐0.100%; however, there was no statistical difference. Greater springiness and chewiness were found in white leg shrimp treated with trehalose and alginate oligosaccharides as compared with water‐soaked samples during frozen storage (Zhang, Cao, et al., 2019; Zhang, Zhao, et al., 2019). Generally, texture attributes in frozen products are improved by adding cryoprotectants in order to partially suppress the mechanical damage caused by ice crystal formation. Polysaccharides are often combined with polar residue of protein to stabilize the muscle tissue (Zhang et al., 2018). Inversely, texture attributes were simultaneously affected by the water content, while lower hardness and shear force were often observed in the samples with higher water content (3.3). In brief, FVP rarely contributes to the texture attributes of freeze‐thawed cultured large yellow croaker and the affecting mechanism needs further investigation.

**TABLE 1:** Changes in the texture of large yellow croaker with different cryoprotectant treatments.

Samples	Hardness (N)	Cohesiveness (Ratio)	Springiness (mm)	Gumminess (N)	Chewiness (mJ)	Shear force (N)
WS	34.16 ± 2.66^b^	0.16 ± 0.01^a^	0.75 ± 0.01^b^	5.14 ± 0.29^b^	3.92 ± 0.23^b^	8.46 ± 1.33^a^
FVP‐0.050%	37.37 ± 2.67^b^	0.17 ± 0.01^a^	0.74 ± 0.01^b^	5.02 ± 0.90^b^	4.35 ± 1.15^b^	9.57 ± 0.67^a^
FVP‐0.075%	38.51 ± 3.44^ab^	0.14 ± 0.00^a^	0.78 ± 0.01^b^	5.35 ± 0.39^b^	4.39 ± 0.22^b^	10.09 ± 0.89^a^
FVP‐0.100%	37.23 ± 4.75^b^	0.16 ± 0.01^a^	0.81 ± 0.04^ab^	5.64 ± 0.95^b^	5.13 ± 0.66^b^	10.16 ± 0.82^a^
CC	47.61 ± 1.63^a^	0.16 ± 0.01^a^	0.87 ± 0.01^a^	8.04 ± 0.01^a^	6.99 ± 0.08^a^	10.09 ± 0.15^a^

*Note*: Results are expressed as mean values ± standard deviation (*n* = 3). Different letters (a and b) represent a significant difference between samples in the same column.

Abbreviations: CC, samples soaked in commercial cryoprotectant; FVP‐0.050%, samples soaked in 0.050% flaxseed polysaccharide gum (FVP) solution; FVP‐0.075%, samples soaked in 0.075% FVP solution; FVP‐0.100%, samples soaked in 0.100% FVP solution; WS, samples soaked in distilled water.

### 3.3 Thawing loss and cooking loss

**FIGURE 3:**
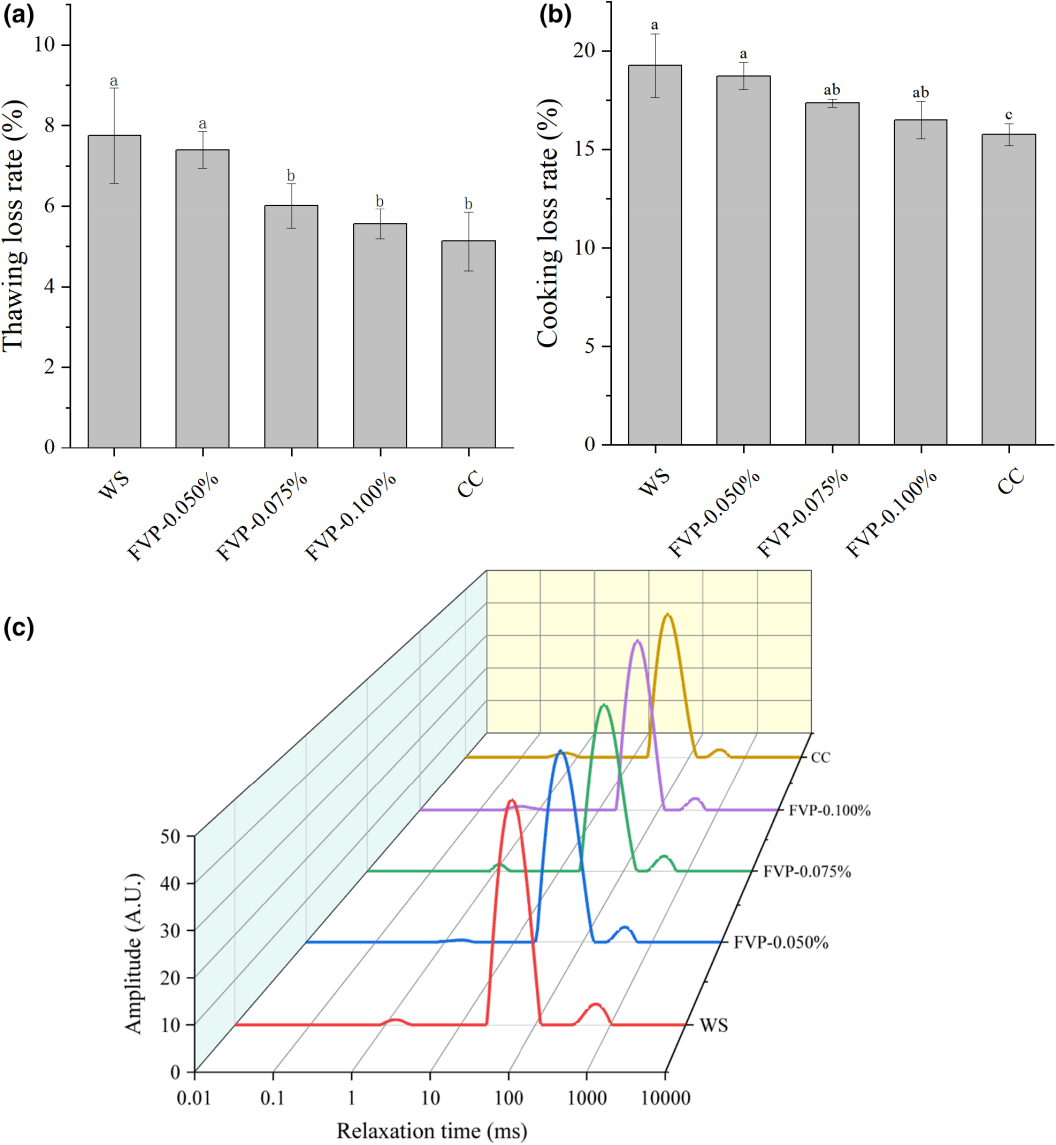
Changes in thawing loss (a), cooking loss (b), and water distribution (c) of large yellow croaker with different cryoprotectant treatments. CC, samples soaked in commercial cryoprotectant; FVP‐0.050%, samples soaked in 0.050% flammulina velutipes polysaccharide (FVP) solution; FVP‐0.075%, samples soaked in 0.075% FVP solution; FVP‐0.100%, samples soaked in 0.100% FVP solution; WS, samples soaked in distilled water. Different letters (a–c) represent the significant difference (*p* < .05) between samples.

Water‐holding capacity plays an important role in frozen products because the moisture loss influences both their commercial and nutritional value, as our results support. The thawing loss and cooking loss of freeze‐thawed large yellow croaker are shown in Figure 3a,b. Variation in thawing loss and cooking loss was revealed to be FVP‐dose dependent, as observed from the decrease in thawing loss and cooking loss with higher FVP addition. There was no significant difference between the WS group and the FVP‐0.050% group, while the FVP‐0.100% group showed a 28.26% and 14.38% reduction in thawing loss and cooking loss, respectively, compared to the WS group. This was close to the effect of the CC group. A similar result was found in frozen peeled shrimp presoaked with carrageenan oligosaccharide and xylooligosaccharide (Zhang, Cao, et al., 2020). Moisture loss in freeze‐thawed muscle is usually caused by mechanical damage induced by the ice crystal growth (Dang et al., 2018), while the free hydroxyl of FVP may reduce the formation of large and irregular ice crystals through binding water molecules (Parvathy & George, 2014). Carillo et al. (2015) used molecular simulations to describe that polysaccharides form a specific capsule structure through amino sugars and uronic acids, which can provide specific hydrogen bonds to the ice surface and inhibit the growth of ice crystals. Additionally, the FVP treatment may retard the denaturation of myofibril protein, and the muscle protein could consequently reabsorb the water molecules during the thawing process.

### 3.4 Water distribution

Low‐field nuclear magnetic resonance (LF‐NMR) analysis was used to further assess the water migration and distribution. The T_2_ relaxation time of the freeze‐thawed large yellow croaker is displayed in Figure 3c. The water in the muscle can be divided into three types according to the T_2_ relaxation time, namely T_2b_ (0–10 ms), T_21_ (10–100 ms), and T_22_ (100–1000 ms). T_2b_ is the bound water tightly combined with the protein molecule, T_21_ is the immobilized water trapped in the thick filament and thin filament of myofibril protein, and T_22_ is the free water present in the outside of muscle fiber (Gudjonsdottir et al., 2015). There was no significant difference found in the T_2b_ of the five presoaked groups, which may be attributable to the intact binding of bound water and protein that remained free from cold injury during this experiment. As for immobilized water, decreased T_21_ was observed with the enhanced FVP concentration, and the FVP‐0.100% group was closely comparable to the CC group. Low T_21_ indicates that the immobilized water was more tightly trapped in the myofibril protein, which thereby reduced the conversion of immobilized water to free water (Zheng et al., 2021). Lan et al. (2020) also found carrageenan oligosaccharides could retard the loss of immobilized water in Pacific white shrimp. The water entrapment hypothesis states that polysaccharides could bind the water molecule more tightly (Zhang, Cao, et al., 2020). Similar results were found in T_22_, with decreases of 0.32%, 0.27%, 0.32%, and 0.29% in FVP‐0.050%, FVP‐0.075%, FVP‐0.1000%, and the CC group, respectively. This reduction in moisture loss during thawing is consistent with the findings of previous studies (3.3), with the 0.100% FVP soaking group exhibiting the best water‐holding capacity. Holt (1991) and Ni et al. (2016) early argued that polysaccharides can reduce the mobility of water and thus alter the growth rate of ice crystals. Also, Regand and Goff (2002) suggested that polysaccharides would impart sufficient microviscosity to the solution to retard the diffusion of water to the crystal interface. In brief, FVP can effectively reduce the thawing loss and cooking loss of freeze‐thawed large yellow croaker by keeping the immobilized water and free water more tightly retained in the muscle tissue.

### 3.5 Flavor analysis

**FIGURE 4:**
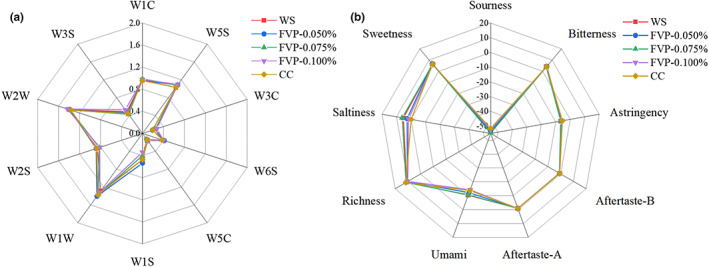
Radar plots of E‐nose (a) and E‐tongue (b) of large yellow croaker with different cryoprotectant treatments. CC, samples soaked in commercial cryoprotectant; FVP‐0.050%, samples soaked in 0.050% flammulina velutipes polysaccharide (FVP) solution; FVP‐0.075%, samples soaked in 0.075% FVP solution; FVP‐0.100%, samples soaked in 0.100% FVP solution; WS, samples soaked in distilled water

Flavor analysis was conducted using an E‐nose system and an E‐tongue system to detect the changes of odor and taste across the different treatment groups. Equipped with 10 sensors, the E‐nose system was used to analyze the changes in volatile compounds; the detailed response values of the different sensors are shown in Figure 4a. In general, almost no significant difference in odor profile was observed for each treatment group. Similar results were reported in lamb meat during the 12‐month frozen storage at −18°C (Rafaella et al., 2013). Only the FVP‐0.050% group showed a slightly higher value in the sensor of W1S (sensitive to methyl) and W1W (sensitive to sulfides, pyrazine), which means that the sample may contain more volatile compounds with methyl groups, inorganic sulfides, and nitrogen oxides. The production and accumulation of inorganic sulfides can lead to undesirable odors in samples, which may relate to protein oxidation and lipid oxidation (Zhang, Yan, et al., 2020). Previous studies have found that carrageenan oligosaccharides play a role in maintaining the initial volatile compounds in frozen‐stored aquatic products (Zhang, Yan, et al., 2020). The level of lipid oxidation and protein oxidation increased slowly during frozen storage, while the frozen period in our study was relatively short, possibly resulting in the similar odor profile (Sveinsdottir et al., 2020).

The electronic tongue (E‐tongue) system used in this study is a multisensor system based on a chemical sensor array combined with a pattern recognition system, equipped with six sensors to detect sweetness, saltiness, richness, umami, aftertaste‐A, aftertaste‐B, sourness, bitterness, and astringency, respectively. Figure 4b shows the taste profile of large yellow croaker with different prefreezing handling. Similar to the results of the odor profile, no significant difference was observed in taste profile, except for slight changes in saltiness and umami. The higher saltiness and umami in the WS group may be attributable to the damage of muscle structure induced by ice crystal growth, which was partly inhibited by the addition of FVP or CC (Qi et al., 2021). Umami partly depends on the content of nucleotides (such as adenosine monophosphate [AMP], guanosine monophosphate [GMP], and inosine monoposphate [IMP]), which was regulated by nucleosidase. Compared to the WS group, FVP and CC may present a higher enzymatic activity of nucleosidase as a result of the cryoprotectant effect, which can be expected to degrade the AMP, GMP, and IMP (Fan et al., 2022). In brief, the addition of FVP barely influences the flavor characteristics of freeze‐thawed large yellow croaker.

### 3.6 Sensory analysis

The sensory evaluation of color, odor, texture, muscle tissue, and compressive score was conducted in the freeze‐thawed cultured large yellow croaker treated with the different soak solutions (Table 2). Significant differences were observed in body color, with the FVP and CC groups each scoring higher than the WS group. The FVP‐0.075% group in particular showed a significant improvement, with a 44.4% increase in the score of the WS group. These results are consistent with those determined by the chroma meter (3.1). Increased body color acceptance was also reported in rainbow trout that were treated with chitosan and pomegranate peel extract before freezing (Berizi et al., 2018). As for muscle tissue, a significant difference was found in the CC group, but not in any of the FVP groups. No significant difference was observed in either texture or flavor profiles, which is again in agreement with the instrumental analysis. A similar odor profile was described in mud shrimp treated with rosemary extract during 12‐week frozen storage (Shi et al., 2019). Odor characteristics depend on small molecule compounds being easily volatilized or running off with the exudate during freezing. In terms of comprehensive sensory score, the FVP group scored higher than the WS group but lower than the CC group.

**TABLE 2:** Changes in sensory evaluation of large yellow croaker with different cryoprotectant treatments.

Sensory attributes	WS	FVP‐0.050%	FVP‐0.075%	FVP‐0.100%	CC
Color	2.66 ± 0.76^a^	3.50 ± 0.45^ab^	3.84 ± 0.95^a^	3.70 ± 0.45^ab^	3.64 ± 0.45^ab^
Odor	3.50 ± 0.63^a^	3.34 ± 0.38^a^	3.50 ± 0.45^b^	3.54 ± 0.55^a^	3.80 ± 0.40^b^
Texture	3.42 ± 0.52^a^	3.63 ± 0.57^a^	3.76 ± 0.54^b^	3.86 ± 0.89^a^	3.80 ± 0.53^b^
Muscular tissue	3.46 ± 0.62^a^	3.56 ± 0.38^a^	3.58 ± 0.38^b^	3.64 ± 0.45^a^	4.44 ± 0.46^a^
Comprehensive score	3.23 ± 0.40^a^	3.51 ± 0.19^ab^	3.63 ± 0.36^ab^	3.69 ± 0.34^ab^	3.92 ± 0.18^a^

*Note*: Results are expressed as mean values ± standard deviation. Different letters (a and b) represent a significant difference between samples in the same row.

Abbreviations: CC, samples soaked in commercial cryoprotectant; FVP‐0.050%, samples soaked in 0.050% flammulina velutipes polysaccharide (FVP) solution; FVP‐0.075%, samples soaked in 0.075% FVP solution; FVP‐0.100%, samples soaked in 0.100% FVP solution; WS, samples soaked in distilled water.

### 3.7 Principal component analysis

**FIGURE 5:**
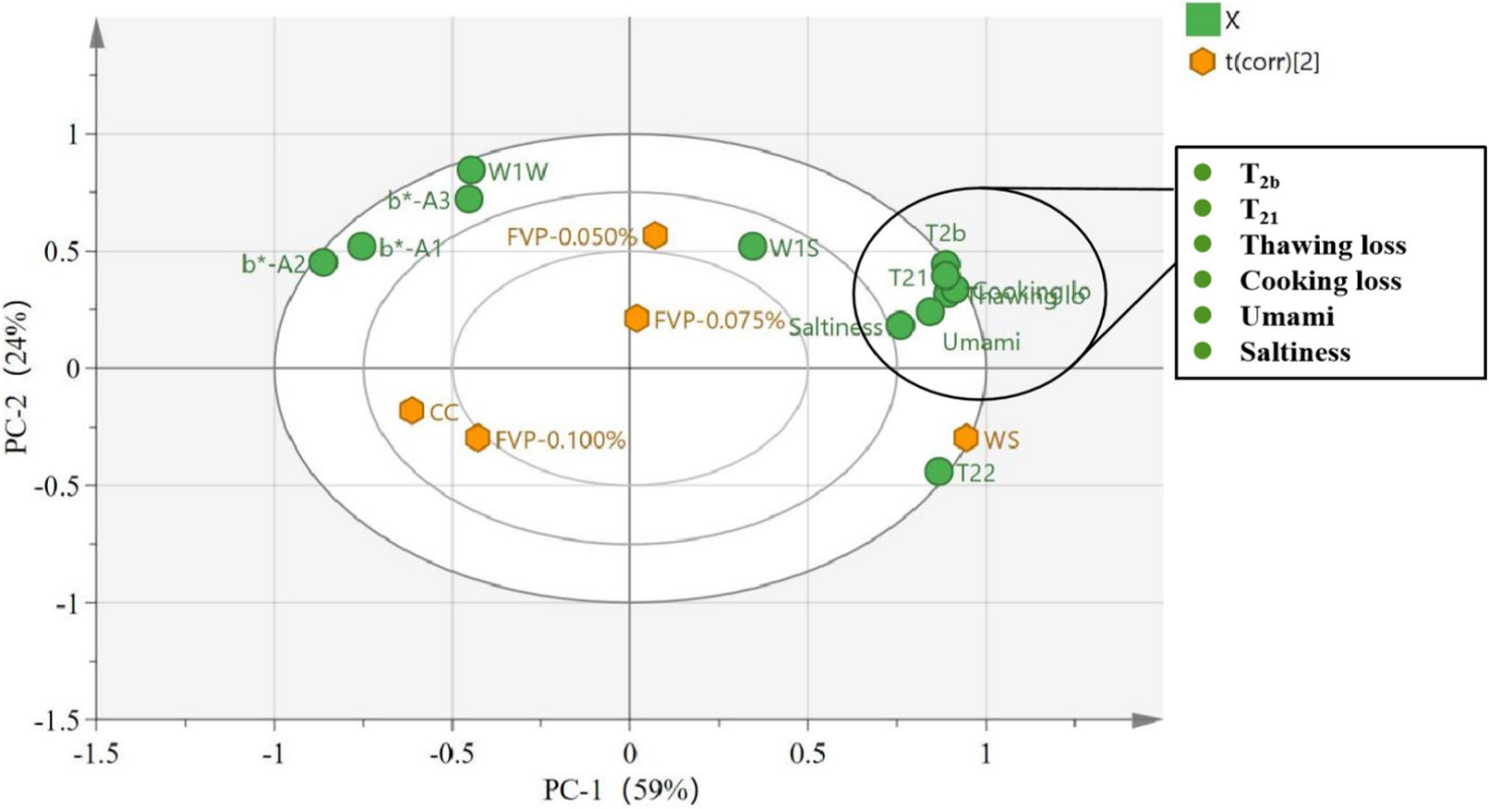
Biplot of principal component analysis (PCA) based on flavor, body color, and water‐holding capacity data of large yellow croaker with different cryoprotectant treatments. CC, samples soaked in commercial cryoprotectant; FVP‐0.050%, samples soaked in 0.050% flammulina velutipes polysaccharide (FVP) solution; FVP‐0.075%, samples soaked in 0.075% FVP solution; FVP‐0.100%, samples soaked in 0.100% FVP solution; WS, samples soaked in distilled water

Principle component analysis (PCA) was performed to further compare the cryoprotective effect on the quality characteristic of freeze‐thawed cultured large yellow croaker (Figure 5). The total variance explained by the first two principal components was 83%, with the first principal component accounting for 59% and the second principal component for 24%. The WS group was distinguished from the FVP‐0.050%, FVP‐0.075%, FVP‐0.100%, and CC groups in first principal component (PC1). It is of interest that the CC and FVP‐0.100% groups show similar results across the experiment, indicating that FVP‐0.100% and CC exhibited an analogous cryoprotective effect. T_22_ proved to be an important indicator leading to an effective distinction between the WS group and the other treatment groups, followed by an increase in umami, saltiness, and moisture loss (cooking loss and thawing loss), as well as a prolongation of T_2b_ and T_21_ relaxation times. The increased T_2_ relaxation time correlated positively with the increase in thawing and cooking losses, with the shorter relaxation time indicating the tight binding of water molecules to the tissue structure. This may be attributable to the conversion of immobile water to free water during freezing induced by the structural changes in myofibril protein. The umami correlated positively with the saltiness, which is consistent with the results in Figure 4b. Additionally, the decreased b* value correlated with the increased water loss, which may be down regulated to the associations of body color and the amount of unbound water which influenced light scattering (Mohammad et al., 2014).

## 4 CONCLUSION

This study investigated the effects of different concentrations of FVP on the water‐holding capacity, muscle texture, body color, odor, and taste of freeze‐thawed large yellow croaker, and compared them with conventional commercial cryoprotectant and water soaking treatments. The results showed that the addition of FVP was effective in alleviating the quality deterioration of freeze‐thawed large yellow croaker, especially in maintaining body color and water‐holding capacity. The FVP treatment had no significant effect on the samples in terms of odor, taste, or improvement of texture. The FVP‐0.100% group had a more pronounced protective effect on water‐holding capacity than the other groups, while the FVP‐0.075% group showed higher b* values. A comprehensive evaluation by sensory and principal component analysis (PCA) revealed that FVP at the 0.100% level showed similar positive effects on the commercial cryoprotectant group. The distribution of ice crystal formation and protein denaturation are critical factors leading to water loss and texture deterioration of aquatic products during the freeze–thaw process; therefore, the mechanism of action of FVP should be further explored in this direction. Concluding, as an alternative type of cryoprotectant, FVP has the potential to significantly reduce the amount of antifreeze added to frozen aquatic products and can avoid to a certain extent the high calories caused by traditional commercial cryoprotectants.

## ACKNOWLEDGEMENT

The study was financially supported by Central Public‐interest Scientific Institution Basal Research Fund, CAFS (2020TD68), Special Research Fund for the National Non‐profit Institutions (East China Sea Fisheries Research Institute) (2021M01), U.S. Agency for National Natural Science Foundation of China (31871872).

## FUNDING INFORMATION

This work was financed by the U.S. Agency for National Natural Science Foundation of China (Grant Number 31871872), Central Public‐interest Scientific Institution Basal Research Fund, CAFS (No. 2020TD68), and the Special Research Fund for the National Non‐profit Institutions (East China Sea Fisheries Research Institute) under the contract number No. 2021M01.

## CONFLICT OF INTEREST

The authors declare that they have no potential conflict of interest.

## Data Availability

Data is contained within the article or supplementary material.
